# Exploring the association between sexual motivation and quality of life in China and the United Kingdom

**DOI:** 10.1371/journal.pone.0293566

**Published:** 2023-12-20

**Authors:** Shijun Zheng, Heather L. Armstrong

**Affiliations:** School of Psychology, University of Southampton, Southampton, United Kingdom; Guangxi Medical University, CHINA

## Abstract

Previous research suggests that different sexual motivations may be associated with different sexual behaviours and levels of sexual satisfaction, and these may vary with cultural differences. As such, sexual motivations and cultural factors might also be associated with quality of life (QoL); however, this has not yet been explored. Using a biopsychosocial approach, this study aimed to explore associations between sexual motivation and quality of life between participants in the United Kingdom and China. 276 participants (143 British, 133 Chinese, mean age = 21.5 years) completed an anonymous online survey including validated measures for sexual motivation (YSEX?-SF) and QoL (WHOQOL-BREF). In multivariable linear regressions, pleasure motivations for sex were associated with increased physical health QoL (aOR = 0.20, 95%CI:.15-.65), while love and commitment motivations were associated with increased psychological health QoL (aOR = 0.13, 95%CI:.01-.43). Both love and commitment motivations (aOR = 0.21, 95%CI:.09-.35) and pleasure motivations (aOR = 0.20, 95%CI:.08-.36) were associated with increased social support QoL. A significant interaction was found between emotional motivations and culture (p = 0.037) such that among individuals from China, emotional motivations (i.e., love and commitment and expression) were positively associated with psychological QoL. This suggests culture may differentially affect how sexual motivations are associated with QoL and warrants further consideration in future research.

## Introduction

Sexual motivation is a psychological concept that considers the driving forces behind sexual activity [1]. In other words, it is the reasons or purposes that individuals engage in sexual behaviours [2].

Using self-determination theory, sexual motivation has been divided into intrinsic and extrinsic aspects [3]. Intrinsic motivation occurs when individuals are self-motivated to engage in pleasurable activities, while extrinsic motivation is driven by external factors such as wanting to please a partner. Engaging in sexual behaviour without any personal intrinsic or extrinsic motivation is referred to as amotivation. The Sexual Motivation scale, developed by Gravel, Pelletier, and Reissing [4], includes these factors and found that self-determined sexual motivation positively correlates with sexual quality of life and overall well-being.

On the other hand, categorizing sexual motivation into only two or three aspects, as per self-determination theory, may overly simplify the wide variety of reasons for which people have sex. In their seminal paper, Meston and Buss [5] identified 237 distinct sexual motivations, which could be grouped into four categories: physical, emotional, goal attainment, and insecurity reasons. Using Meston and Buss’s YSEX? Scale [5] may provide a more nuanced understanding of how sexual behaviour are associated with overall well-being [6, 7].

### Quality of life (QoL)

The World Health Organization [8] defines QoL as an individual’s thoughts or perspectives of their position in life in relation to the culture and value system in which they reside, as well as one’s own desires, expectations, standards, and worries. QoL has been studied extensively in health psychology, particularly in relation to conditions such as breast cancer and autism spectrum disorder [9, 10]. Studies have also shown that sexual function is associated with QoL in people with these conditions [11, 12], with Carreira et al. [13] finding that women with breast cancer had both low sexual function and lower QoL.

Additionally, mindfulness-based stress reduction (MBSR), a psychological intervention designed to improve QoL, has shown promise in improving sexual QoL among menopausal women [14]. MBSR has also been associated with reduced sexual distress among women with low sexual desire [15]. Finally, it has been suggested that using a biopsychosocial approach is beneficial when considering and studying QoL to better understand the subjective experience of individuals’ overall well-being [16].

### Love, pleasure, and reproduction motivations for sex

While Meston and Buss found 237 different reasons for having sex, the most commonly reported motivations were for emotional/love reasons and for pleasure [5, 17]. Sex for reproduction, while not as commonly reported, is also a significant driver of human sexual behaviour [17].

Love and commitment motivations for sex may lead to increased QoL in several ways. For example, among a sample of 128 Israeli couples, having intimacy motivations for sex was associated with sexual satisfaction and sexual intimacy among both men and women, and with orgasmic responsivity for women [18]. Likewise, in a series of studies by Muise and colleagues [19], pursuing sex for approach goals such as to enhance intimacy was associated with increased sexual and relationship satisfaction both in the short and longer terms. Engaging in a romantic relationship and spending time with a partner can also increase the sense of love and well-being [20, 21] and feeling loved is a form of social support which contributes to maintaining a romantic relationship [22]. As such, love and commitment motivations may be positively associated with several aspects of QoL including physical, emotional, and social well-being.

Secondly, sexual pleasure is important for most people and understanding it is essential for sexual well-being [23, 24]. Sexual pleasure during sex is well established to lead to higher sexual satisfaction and may also be associated with improved quality of life [25, 26]. However, others have suggested that focusing only on having sex for pleasure might lead to individuals deprioritising or neglecting other aspects of sexual health, perhaps leading to things like reduced condom use [27] which could lead to decreased well-being for some. As such, the association between pleasure as a sexual motivation and physical QoL needs further exploration.

Finally, people who have sex for reproduction proposes may also experience different levels of QoL. For example, couples who are planning for pregnancy may make other healthier behaviour choices such as quitting or reducing alcohol and smoking [28] as pre-pregnancy preparation can lead to improvement in parental health as well as infant health and reduced chance of pregnancy loss [29]. This may lead to overall improved physical health QoL. Additionally, when both members of a couple have shared reproductive motivations, this can lead to improved relationship outcomes and better psychological well-being [30, 31]. Therefore, studying reproduction as one aspect of sexual motivation can also contribute to increased understanding of QoL.

### Cultural differences

To better understand sexual motivation, cultural differences must also be considered. Laan et al. [32] highlighted significant differences in the way people from different cultures express their views and opinions about sexuality. For example, sexual attitudes and education are substantially different in China and the UK. In China, sex education mostly focuses on preventing HIV, STIs, and casual sex, while sexual pleasure and expression are not discussed [33]. While Chinese parents generally support teaching children about sexuality, very few actually have "the talk" with their children [34]. In contrast, sexual health educators in the United Kingdom are more likely to share more comprehensive knowledge with students including issues of gender equality and sexual safety online [35]. Thus, cultural differences might influence individuals’ sexual motivation and well-being.

### Current study

This study aims to investigate the relationship between sexual motivation and QoL from a biopsychosocial perspective, and to explore cultural differences in motivation and QoL between China and the UK. Previous research has not yet examined the correlation between sexual motivation and QoL. Additionally, it is unclear whether cultural factors are differentially related to the association between sexual motivation and QoL. The study proposes two hypotheses based on this information.

The first hypothesis is that higher scores on "love and commitment", "reproduction", and "pleasure" motivations will be associated with higher scores on physical health, psychological health, and social support quality of life.

The second hypothesis is that country of residence and birth will moderate the associations between "emotional motivation" and physical health, psychological health, and social support quality of life scores.

## Methods

### Sample and recruitment

Inclusion criteria for this anonymous, online survey included living in China or the UK, being aged 18 and above, and being able to complete the questionnaire in English. Participants were recruited via social media: Twitter for UK residents and WeChat, Weibo, and Xiaohongshu for Chinese residents. Additionally, an advertisement was placed on the undergraduate student subject pool at the University of Southampton. Students who completed the study via the subject pool received two course credits; no other compensation was offered.

### Procedure

Data was collected from March 14 to June 3, 2022. Interested individuals clicked on the survey link in the advertisement and were taken to the survey which was hosted on iSurvey for UK participants and Wengjuanxing for Chinese participants. After reading the participant information sheet, participants gave informed consent by checking a box at the bottom of the page. They were then presented with demographic questions and validated measures for sexual motivation and quality of life. A debrief form with researcher contact information was provided at the end of the survey.

### Measures

#### Demographics

Demographic questions included age, gender, sexual orientation, ethnicity, education, employment, country of birth, and country of current residence. Only participants born and living in the same country were included in the analytic sample, to minimise additional influences on cultural attitudes or values [36].

#### Sexual motivation

The Why Have Sex Short-Form Questionnaire (YSEX?-SF) [17] was used to measured sexual motivation. The scale includes 28 items and participants rate their level of motivation for each item on a 5-point Likert scale. The 28 items are grouped into 14 factors, 3 of which are considered in this analysis (i.e., pleasure, reproduction, and love & commitment). An additional factor, "emotional motivation” comprised of “love and commitment” and “expression” motivations [5] was also included in the analysis.

#### Quality of life

To assess quality of life, the WHO Quality of Life BREF (WHOQOL-BREF) [37] was used. The WHOQOL-BREF is a 26-item scale which has been validated cross culturally [38] and is divided into four subdomains: physical health, mental health, social support, and environment-related health quality of life. Xia et al. and Skevington et al. found good internal consistency (Cronbach’s α = .89 and .88, respectively) for Chinese and United Kingdom samples [39, 40]. For this analysis, in line with the biopsychosocial model, only the physical, psychological, and social health QoL scores were used.

### Data analysis

Data were cleaned and validated before analysis in SPSS version 28, 2022. Poor quality data were deleted (i.e., where participants selected the same answer to each question). To address Hypothesis 1, univariable linear regressions were used to test the association between each independent variable (i.e., love & commitment, pleasure, and reproduction motivations) and each dependent variable (i.e., physical, psychological, and social health QoL). Independent variables which were significantly (p < .05) or potentially significantly (p < .20) [41] associated with each DV were retained for testing in multivariable linear analysis. In order to detect an effect size of f^2^≥0.05, with α = 0.05 and β = 0.80, in a multivariable linear regression with three independent variables, a minimum sample size of 222 is needed. To examine Hypothesis 2, three moderation analyses were run in SPSS PROCESS Marco version 4. Emotional motivation score was considered as the independent variable, physical, psychological and social health QoL were considered in separate analyses as dependent variables, and country of birth and residence (i.e., China or the UK) was considered as the moderator. Significance was considered at p < .05.

## Results

### Demographic characteristics

276 participants completed the survey and are included in the analysis. Most were female (76.8%) and age ranged from 18 to 48 years (M = 21.5, SD = 3.39). Participants were roughly split between the United Kingdom (n = 143, 51.8%) and China (n = 133, 48.2%). Likewise, most participants identified as White (44.9%) or Asian (50%). Most participants (75.7%) were full-time students and most (68.8%) identified as straight. Full demographics are presented in Table 1.

**Table 1:** Demographic characteristics of participants (n = 276).

Characteristic	n	%
Country of birth and residence		
United Kingdom	143	51.8
China	133	48.2
Ethnicity		
White	124	44.9
Black or Black British	5	1.8
Asian	138	50
Middle Eastern or North African	1	0.4
Some other race/ethnicity	8	2.9
Gender		
Female	212	76.8
Male	60	21.7
I use something else for my gender	4	1.4
Work Status		
Employed	53	19.2
Out of work and looking for work	6	2.2
A homemaker	5	1.8
A student and not looking for work	209	75.7
Unable to work	3	1.1
Education		
No schooling completed	16	5.8
High school graduate diploma or the equivalent	155	56.2
Bachelor’s degree	74	26.8
Master’s degree	31	11.2
Sexual Orientation		
Heterosexual	190	68.8
Gay/Lesbian	15	5.4
Bisexual	55	19.9
Pansexual	7	2.5
Asexual	3	1.1
Rather not say	6	2.2

### Hypothesis 1

A histogram indicated normal distribution for physical and psychological health QoL, and slightly negative skewness for social support QoL, which was acceptable based on the residual plots (see S1 Appendix) [42]. The mean and standard deviations of all study variables are reported in Table 2.

**Table 2:** Descriptive statistics for all study variables for the whole sample and by country.

Variable	Total (n = 276)		United Kingdom (n = 143)		China (n = 133)	
	M	SD	M	SD	M	SD
Physical health QoL	25.13	4.04	26.10	4.13	24.10	3.67
Psychological health QoL	19.24	4.01	18.98	4.31	19.52	3.65
Social support QoL	10.67	2.32	11.20	2.28	10.10	2.23
Love and commitment	6.78	2.25	6.97	2.18	6.56	2.31
Pleasure	6.75	2.05	7.69	1.54	5.75	2.06
Reproduction	2.69	1.45	2.12	0.61	3.31	1.80
Emotional	11.42	3.61	11.38	3.43	11.46	3.81

Table 3 displays the results of nine simple linear regression tests, indicating the linear relationships between the independent (love and commitment motivation, pleasure motivation, and reproduction motivation) and dependent variables (physical health QoL, psychological health QoL, and social support QoL). Results showed that love and commitment motivation and pleasure motivation were significantly positively associated with physical health QoL and social support QoL while love and commitment motivation was significantly positively associated with psychological health QoL.

**Table 3:** Simple linear regressions between independent and dependent variables.

	Physical Health QoL		Psychological Health QoL		Social Support QoL	
	OR	p	OR	p	OR	p
Love and Commitment	0.12	.043	0.13	.027	0.28	< .001
Pleasure	0.22	< .001	0.05	.42	.029	< .001
Reproduction	-0.08	.207	0.09	.145	-0.10	0.98

Table 4 shows the results of three multiple linear regressions. Greater pleasure motivation scores (aOR = 0.20, 95% confidence interval (CI): .15-.65, p = .002) were independently associated with better physical health QoL, F(2, 273) = 7.08, p = .001, R^2^ = .05, R^2^_adjusted_ = .04. Higher love and commitment motivation scores (aOR = 0.13, 95%CI:.01-.43, p = .038) were independently associated with increased psychological health QoL, F(2, 273) = 3.26, p = .04, R^2^ = .02, R^2^_adjusted_ = .02. Finally, higher love and commitment motivation (aOR = 0.21, 95%CI:.09-.35, p < .001) and higher pleasure motivation (aOR = 0.20, 95%CI:.08-.36, p = .002) scores were each positively associated with social support QoL, F(3, 272) = 13.14, p < .001, R^2^ = .13, R^2^_adjusted_ = .12.

**Table 4:** Multiple linear regressions between independent and dependent variables.

	Physical Health QoL			Psychological Health QoL			Social Support QoL		
	aOR	95%CI	p	aOR	95%CI	p	aOR	95%CI	p
Love and Commitment	0.04	-.15,.30	.502	0.13	.01, .43	.038	.21	.09, .35	< .001
Pleasure	0.20	.15, .65	.002	not included			0.20	.08, .36	.002
Reproduction	not included			0.08	-.12, .54	.213	-.10	-.34, .02	.088

### Hypothesis 2

**Fig 1:**
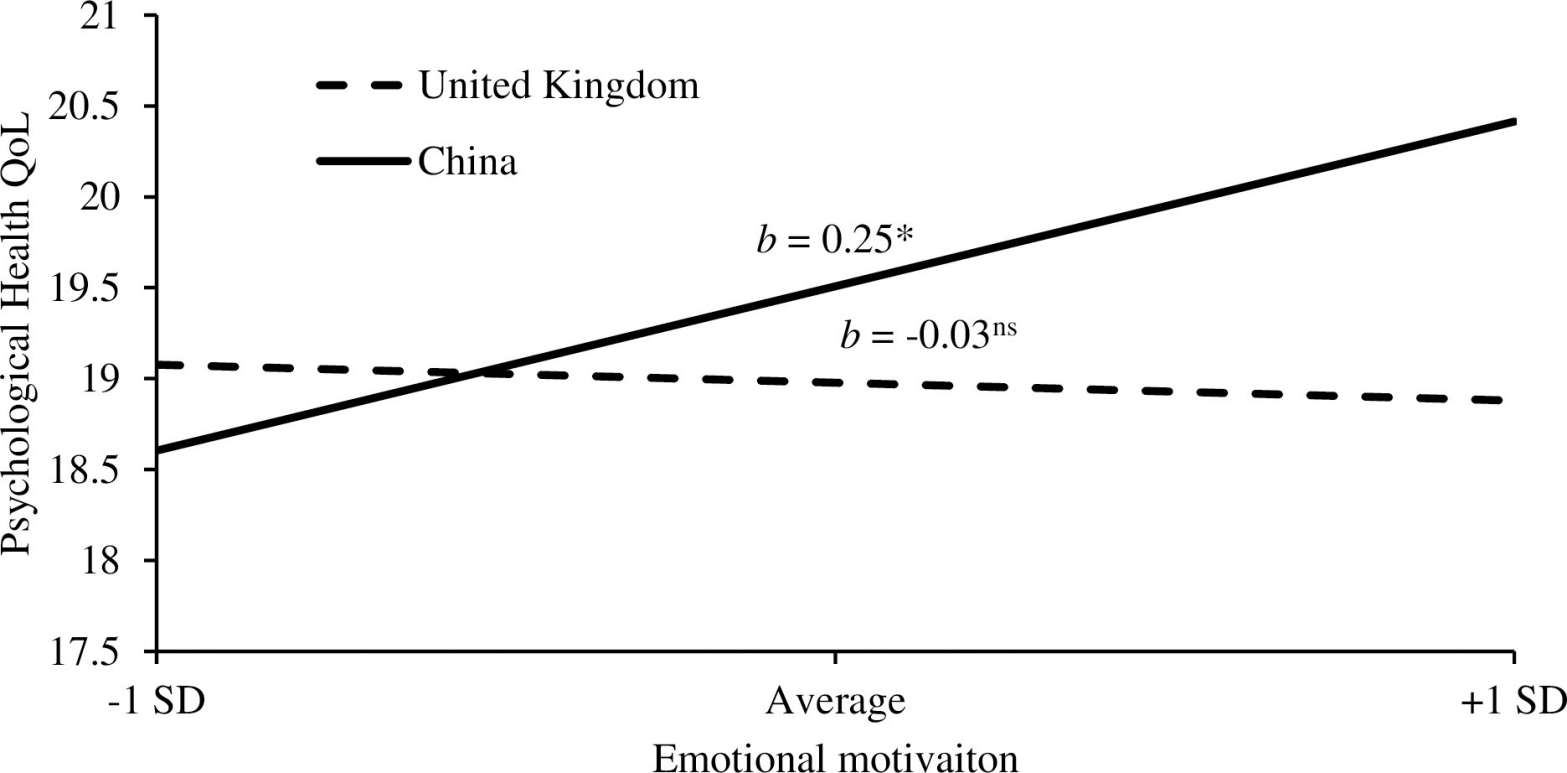
Cultural differences in the effect of emotional motivation on psychological health QoL. Note. *p < .05. ns = no significance. SD = standard deviation.

Using the PROCESS macro version 4, a moderation analysis showed an interaction of cultural background and emotional motivation on psychological health QoL which explained 3% of the total variability (Table 5). Chinese cultural background significantly moderated the relationship between emotional motivation and psychological health QoL (Fig 1). Individuals from China with higher emotional motivation had higher psychological health QoL (b = .25, 95%CI: .07-.43, p = .006). However, no significant moderation was found for individuals from the United Kingdom (b = -.03, 95%CI: -.22-.16, p = .778).

**Table 5:** Moderation analysis of predictors of psychological health QoL.

	b	SE B	t	p	R^2^
Emotional Motivation	-0.31	0.21	-1.43	.155	
Culture	0.53	0.48	1.11	.267	
Emotional Motivation x Culture	0.28	0.13	2.09	.037	.03

The moderation analysis did not find significant interactions between cultural background and emotional motivation for social support QoL (b = .06, 95%CI: -.08-.21, p = .402) or for physical health QoL (b = .22, 95%CI: -.03-.48, p = .090).

## Discussion

This research investigated the relationships between sexual motivation and three different types of QoL; it further explored cultural background as a moderator for the association between emotional motivations and psychological health QoL. Key findings include positive associations between pleasure motivation and physical health QoL, and love and commitment motivation and psychological health. Thirdly, pleasure motivation and love and commitment motivation were positively associated with social support QoL. Chinese cultural heritage moderated the association between psychological health QoL and emotional motivation such that individuals from China with higher emotional motivation for sex had higher psychological health QoL whereas no difference was found among those from the UK.

### Sexual motivation & QoL

The current study found that having sex for pleasure purposes was positively associated with physical health QoL. A possible explanation may be that individuals who value physical pleasure, and are therefore more likely to pursue sex for pleasure reasons, may also place more value on their physical health and body image. Additionally, past research also suggests associations between increased sexual pleasure, physical health, and body image [43–45]. Further, evidence suggests that physical activity and exercise may improve erectile function [46] which could also lead to improved sexual pleasure and satisfaction and sexual intercourse itself may be considered as good exercise [47]. Overall, the relationship between having sex for pleasure and improved physical health QoL is likely to be bidirectional and may also be associated with improved physical health and body image, although the nature of any such relationship remains to be explored.

Secondly, having sex for love and commitment reasons was associated with better psychological health QoL, which is in line with studies by Brunell and Webster [48] who found that love and commitment in romantic relationships are linked to better sexual satisfaction and psychological health. Accordingly, Mostova, Stolarski, and Matthews [49] found that expressing and receiving love in preferred ways, such as physical touch, can increase sexual satisfaction and overall psychological well-being [50]. Muise and Impett also found that those with higher communal strength, that is people who are motivated to respond unconditionally to their partners’ sexual requirement, are more likely to satisfy their partner’s sexual needs, leading to increased relationship and psychological well-being [51]. Therefore, having sex for love and commitment motivations can improve psychological health QoL by allowing people to meet their own and their partner’s sexual needs, leading to better overall psychological well-being.

Finally, love and commitment motivations and pleasure motivations for sex were associated with improved social support QoL. There are at least two possible explanations. First, sex may enhance the bond between partners and increase satisfaction with social interactions, which can lead to stronger social support. For example, Kapsaridi and Charvoz’s review found that men in strong romantic relationships had stronger social support, helping them manage stress and other relationships [52]. Second, according to self-determination theory, engaging in sex for pleasure is an intrinsic motivation that satisfies individual needs and increases relationship well-being [53]. Romantic partners may have sex for different reasons, such as fulfilling sexual needs and enhancing relationship satisfaction. Self-determined individuals are more likely to be attached to their partners and fulfil their sexual needs, while those motivated by love and commitment may prioritize relationship satisfaction [54, 55]. Overall, individuals with love and commitment motivations and pleasure motivations may improve their social well-being through sexual fulfilment and enhanced relationship satisfaction, leading to better social relationships.

### Culture as a moderator of emotional motivation and psychological health QoL

Our study found a moderating effect of culture such that Chinese participants showed a significant positive association between emotional motivation and psychological health QoL, while participants from the United Kingdom did not. While past research has found no difference in sexual motivation between Chinese and Western participants [56], cultural barriers experienced by Chinese individuals may explain the stronger association between emotional motivation for sex and psychological health, since individuals in China may have less opportunity to engage with formal sexual education programmes [57] and may have restricted access to certain literature and other education resources. Consequently, Chinese individuals may rely on family or cultural sources to learn about sexuality which may result in a greater emphasis on emotional factors, such as love and connection, as motivators for sexual activity [58].

In contrast, participants from the United Kingdom did not demonstrate a relationship between emotional motivation and QoL. This is consistent with work by Higgins et al. which found that although some of the socio-historical changes are similar, the fundamental attitudes toward sexuality in the Chinese and United Kingdom samples have significantly different emphasis and preferences [59]. Additionally, people from the UK may not consider having sex for emotional purposes as a primary way to regulate their psychological well-being [60, 61]. For instance, compared to sex education in China, sex education in the United Kingdom teaches more about seeking sexual pleasure and satisfaction, rather than only an emotion-based approach to sex, which may lead to a positive association between mental health and physical pleasure [62]. Therefore, the significant association between emotional motivation and psychological QoL in the Chinese participants may be due to cultural differences, differences in sexual education, and different attitudes towards sexual health.

### Reproduction motivations and QoL

Unexpectedly, reproduction motivation was not associated with physical, psychological, or social QoL. This may be because our participants were mainly young university students who may not prioritize or want to have children, or who may fear childbirth leading to declined motivation to have sex for reproductive purposes [63]. Additionally, university students often experience high levels of stress, which could further discourage sexual activity for reproduction [64]. Therefore, our findings suggest that reproduction motivation may not be significantly associated with biopsychosocial quality of life in young university students; however, this should be further explored in older and more diverse samples.

### Strengths and limitations

The present study compares Chinese and United Kingdom samples, considers cultural factors, and provides a diverse viewpoint beyond solely a Western focus which may make it relevant to a wider range of people. As Chinese immigrants are one of the fastest-growing groups in the United Kingdom [65], these findings may help sexual health clinicians working in the UK to better understand the association between sexual motivation and QoL among cultures, leading to more appropriate sexual health interventions for diverse cultural communities.

This study also had several limitations. Firstly, the questionnaire for the Chinese sample was also in English, which may have led to misunderstandings and may have influenced the findings. Additionally, while the sample was balanced between participants from the UK and China, the sample was predominantly made of young university students. As such, their motivations for sex may differ from peers not in university and from older adults, particular with respect to reproductive motivations.

### Future research

Results of this study suggest several directions for future research. Firstly, including participants from different age groups may provide a more comprehensive understanding of how sexual behaviours vary across different ages and across the lifespan. Secondly, developing or validating a YSEX? Questionnaire specific to the Chinese population could shed light on the reasons behind sexual behaviours under strict sexual policies, particularly among different subgroups such as men and women of different ages.

Additionally, future studies could benefit from studying other countries with significant immigrant populations, such as Australia, Canada, and the United States. Comparing findings across these diverse cultural contexts could help to better understand how cultural differences impact sexual motivation and quality of life, and how the interactions between immigrants and non-immigrants shape sexual attitudes and behaviours [66]. For example, investigating the effects of policy transitions, such as the shift from a White Australia policy to multiculturalism, could provide valuable insights into the impact of policy changes on sexual health [67, 68].

## Conclusion

This is the first research to examine the associations between sexual motivation and QoL from a biopsychosocial viewpoint, as well as to consider how these relationships are influenced by cultural background. Findings suggest that sexual motivations for love and pleasure are positively associated with mental and physical health QoL, respectively, and both motivations are linked to higher social support QoL. Notably, emotional motivation was positively associated with psychological health QoL in the Chinese sample only. These results may have implications for sex education programmes and sexual health interventions and provides an initial understanding of the associations between sexual motivation and QoL.

## Supporting information

S1 Appendix. Histograms and residual plot (DOCX)
