# Supplementary material for: Exploring the association between sexual motivation and quality of life in China and the United Kingdom
Source: PLoS One. 2023 Dec 20;18(12):e0293566. doi: 10.1371/journal.pone.0293566 (PMC10732451; doi:10.1371/journal.pone.0293566)

Exploring the Association between Sexual Motivation and Quality of Life in China and the United Kingdom

**S1: Appendix A**

**Histograms and Residual Plot**

This appendix consists of three histograms regarding the normality of psychological health QoL, physical health QoL and social support QoL scores. Additional, there is also a residual plot regarding the multiple linear regression between love and commitment and pleasure motivations and social support QoL.


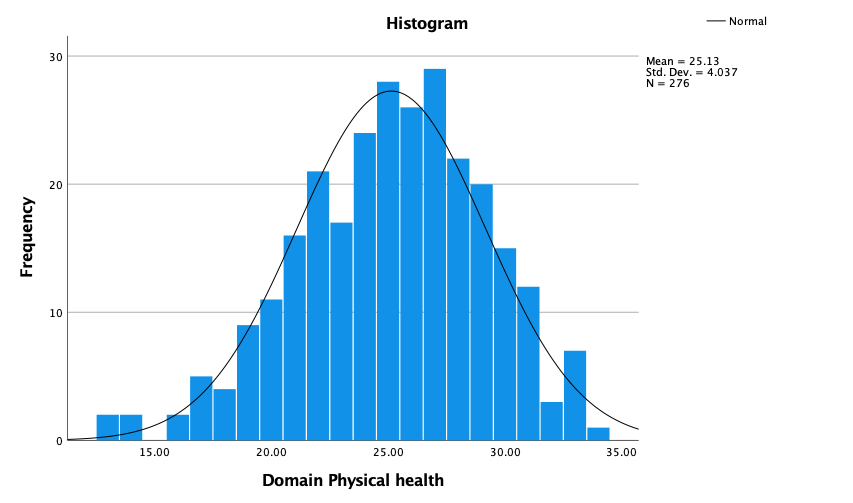

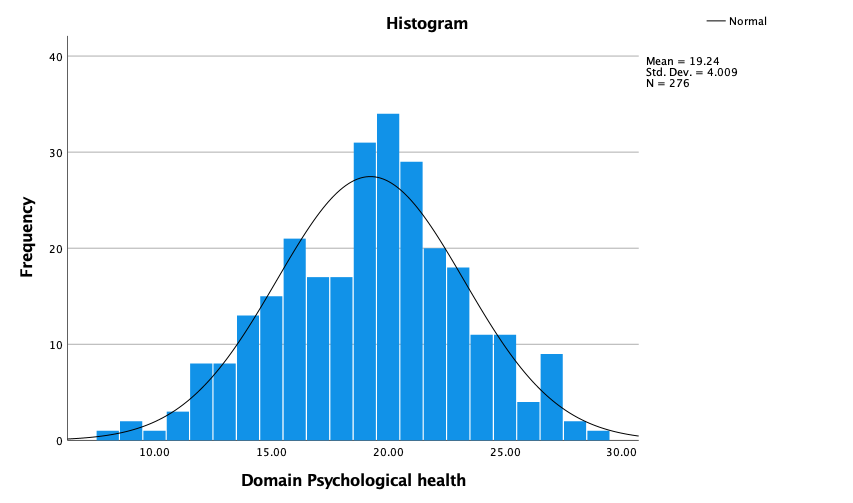

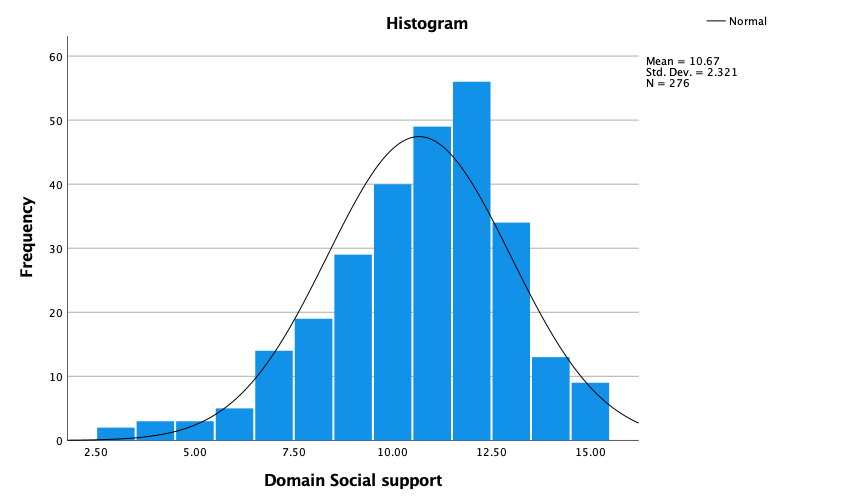

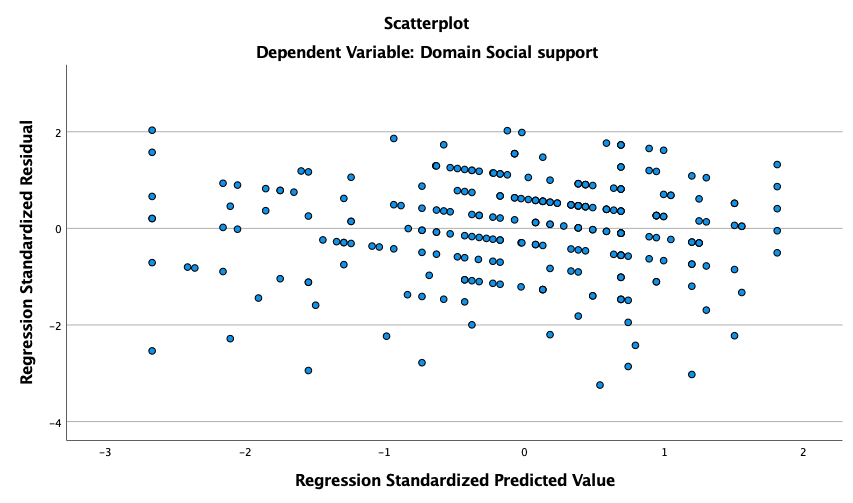

Supplement: S1 Appendix. Histograms and residual plot — (DOCX) [file pone.0293566.s001.docx]
